# Association of two geriatric treatment systems with anti-osteoporotic drug treatment and second hip fracture in patients with an index hip fracture: retrospective cohort study

**DOI:** 10.1186/s12877-024-04989-0

**Published:** 2024-05-04

**Authors:** Kilian Rapp, Patrick Roigk, Clemens Becker, Chris Todd, Martin Rehm, Dietrich Rothenbacher, Claudia Konnopka, Hans-Helmut König, Thomas Friess, Gisela Büchele

**Affiliations:** 1 Department of Clinical Gerontology, Robert-Bosch-Hospital, Stuttgart, Germany; 2 Unit Digitale Geriatrie, Universtiätsklinikum Heidelberg, Heidelberg, Germany; 3 School of Health Sciences, Faculty of Biology, Medicine and Health, The University of Manchester, Manchester, M13 9PL UK; 4 Manchester University NHS Foundation Trust, Manchester, M13 9WL UK; 5 Institute of Epidemiology and Medical Biometry, Ulm University, Helmholtzstr. 22, 89081 Ulm, Germany; 6 Center for Trauma Research, Ulm University, Ulm, Germany; 7 Department of Health Economics and Health Services Research, University Medical Center Hamburg-Eppendorf, Hamburg, Germany; 8 AUC - Akademie der Unfallchirurgie GmbH, Wilhelm-Hale-Straße 46B, Munich, 80639 Germany

**Keywords:** Hip fracture, Second fracture, Geriatric, Rehabilitation, Anti-osteoporotic drugs

## Abstract

**Background:**

In Germany, geriatricians deliver acute geriatric care during acute hospital stay and post-acute rehabilitation after transfer to a rehabilitation clinic. The rate patients receive acute geriatric care (AGC) or are transferred to post-acute rehabilitation (TPR) differs between hospitals. This study analyses the association between the two geriatric treatment systems (AGC, TPR) and second hip fracture in patients following an index hip fracture.

**Methods:**

Nationwide health insurance data are used to identify the rate of AGC and TPR per hospital following hip fracture surgery in patients aged ≥ 80 years. Outcomes are a second hip fracture after surgery or after discharge within 180 or 360 days and new specific anti-osteoporotic drugs. Cox proportional hazard models and generalised linear models are applied.

**Results:**

Data from 29,096 hip fracture patients from 652 hospitals were analysed. AGC and TPR are not associated with second hip fracture when follow-up started after surgery. However, during the first months after discharge patients from hospitals with no AGC or low rates of TPR have higher rates of second hip fracture than patients from hospitals with high rates of AGC or high rates of TPR (Hazard Ratio (95% CI) 1.35 (1.01–1.80) or 1.35 (1.03–1.79), respectively). Lower rates of AGC are associated with lower probabilities of new prescriptions of specific anti-osteoporotic drugs.

**Conclusions:**

Our study suggests beneficial relationships of geriatric treatment after hip fracture with a) the risk of second hip fractures during the first months after discharge and b) an improvement of anti-osteoporotic drug treatment.

**Supplementary Information:**

The online version contains supplementary material available at 10.1186/s12877-024-04989-0.

## Background

A fracture is a strong risk factor for future fractures. There is good evidence that the risk of a second fracture is particularly high immediately after the first fracture and declines thereafter [1, 2]. Reasons for this imminent fracture risk may be muscle weakness and impaired coordination due to immobilisation, post-operative delirium, changes in medication regimens with increases in fall-risk-increasing drugs (FRIDs), or a new environment due to institutionalisation [3–5]. Therefore, one of the aims of fracture treatment should be the prevention of second fractures. Since most fractures in older people are caused by falls and/or low bone mass, reduction of fall risk and improvement of bone mass seem obvious measures to reduce the imminent fracture risk [6].

Hip fractures amongst older people are often low-impact fragility fractures occuring in frail people with comorbidities. Therefore, geriatricians are increasingly involved in the treatment of patients with hip fracture [7], for example in terms of orthogeriatric co-management during the acute hospitalisation or post-acute geriatric rehabilitation after ‘transfer to a post-acute rehabilitation’ clinic. Regaining safe mobility and treatment of osteoporosis are key elements of geriatric treatment in these hip fracture patients. However, there is no evidence that the implementation of geriatric treatment standards reduces the incidence of second fractures. Hawley, for example, reports an observational pre-post analysis of a large dataset from British hospitals with orthogeriatric co-management or fracture liaison service and observes reduction in mortality in hip fracture patients over 10 years but no reduction of second hip fractures [8]. Reviews and meta-analyses which evaluate the effects of orthogeriatric co-management report outcomes like mortality, length of stay, or delirium but do not mention second fractures as outcome [9–13]. Only the review by van Camp et al. [14] reports second fractures as an outcome concluding that the evidence on prevention of subsequent fractures is scarce and inconclusive as it is based on only two small studies [15, 16].

In Germany, geriatricians are involved in the care of patients with hip fracture either directly after surgery in form of acute geriatric care (AGC) or after a ‘transfer to a post-acute geriatric rehabilitation’ clinic (TPR). AGC takes place during the acute hospital stay as orthogeriatric co-management in cooperation with orthopaedic surgeons. Geriatricians are exclusively in charge of the treatment in post-acute geriatric rehabilitation units.

In the past, patients with hip fracture were offered either AGC or TPR depending upon the federal state in which they were hospitalised [17]. Since 2012 circumstances have changed considerably. Hospitals offering exclusively ‘post-acute geriatric rehabilitation’ established AGC, whilst hospitals from federal states previously offering only AGC developed post-acute geriatric rehabilitation clinics [18]. During this transition period hip fracture patients may have received either I) only AGC or II) only TPR or III) both AGC and TPR. However, not all patients actually received one or even both types of geriatric treatment. Reasons were, that the infrastructure of the clinic did not allow offering AGC to all patients, that patients’ functional status did not qualify for AGC or for rehabilitation in a post-acute clinic, or that geriatric rehabilitation clinics were not available in the vicinity. Therefore, the percentages of AGC or TPR were considerably lower than 100% and differed from clinic to clinic.

We used this transition period in Germany to analyse the association between the two geriatric treatment systems on the initiation of anti-osteoporotic drugs—as a mediating factor—and on incidence of a second hip fracture in patients with an index hip fracture.

## Methods

### Study design, data source and study population

The basic dataset for this retrospective cohort study consists of patients aged ≥ 80 years admitted to hospitals with a hip fracture between the index dates of 01.01.2015 and 30.06.2016 in Germany and insured by the “Allgemeine Ortskrankenkasse” (AOK) insurance company. AOK is Germany’s largest health insurance company and covers nearly one-third of Germany’s 82.5 million population. Patient-related health insurance claims data were provided by the scientific institute of the AOK (“Wissenschaftliches Institut der AOK”, WIdO) in de facto anonymised manner. After exclusion of patients due to methodological reasons the final ‘Analysis set’ consisted of 29,096 patients. Reasons for exclusion were a) an insurance gap in the period between 365 days prior to hospital admission till the end of the individual follow-up period after first surgical treatment (46 patients) and b) a previous fracture in the 180 days before index hospital admission (264 patients).

Hip fractures were identified using hospital admission diagnostic codes S72.0 and S72.1 (ICD-10) in combination with a procedure code for surgery since nearly all hip fractures are treated by surgery. The term ‘index hip fracture’ refers to the first hip fracture identified as suffered by an individual during the study period. Analyses were restricted to patients aged ≥ 80 years since they are by definition regarded as geriatric patients due to their age-specific vulnerability, which means increased risks of complications, chronic conditions, and loss of autonomy [19]. The assumption was that all patients aged ≥ 80 years should benefit from geriatric care.

Acute geriatric care (AGC) can be identified in insurance health claims data by the German procedure classification code ‘OPS8-550’. This represents a complex treatment of early rehabilitation lasting at least 14 days (see Table 1 for details). In patients with hip fracture, AGC begins soon after surgery and can be delivered as orthogeriatric co-management on an orthopedic or a geriatric unit. Some orthopedic units, which do not offer AGC, transfer hip fracture patients early after surgery to other hospitals which provide AGC. Hospitals with such transfer exceeding 5% of their hip fracture patients were excluded from the analyses to avoid a) misclassification of exposure and b) selection bias of transferred (and not transferred) patients (12,766 patients from 360 hospitals).

**Table 1:** Characteristics of the two geriatric treatment systems for hip fracture patients in Germany

	**Acute geriatric care (AGC)**	**Post-acute rehabilitation**
Start	Soon after surgery	Usually 7 to 14 days after surgery
Time period	At least 14 days	3 weeks, sometimes extended by 1 or 2 weeks
Orthogeriatric co-management	Yes	no
Procedure code	OPS8-550	–
Place	Acute clinic The orthogeriatric comanagement is delivered on an orthopedic or a geriatric unit. In Germany, common models are either shared responsibility on an orthogeriatric unit or a geriatric liaison service on the orthopedic unit with an early transfer to a geriatric unit	Post-acute rehabilitation clinic
Transfer to the treatment place required	no	yes
Application at and approval by the health insurance required	no	yes
Multidisciplinary geriatric team headed by a geriatrician	yes	yes
Comprehensive geriatric assessment	yes	yes
Treatment	At least 20 units of therapy usually delivered as individual therapies within 14 days	2–4 individual or group therapies per day over 3 or more weeks

After the acute hospital stay, patients can be transferred to a post-acute rehabilitation clinic (TPR). This information is recorded in routine hospital datasets permitting calculation of the rate of TPR per hospital. Post-acute geriatric rehabilitation needs upfront approval by the health insurer, is delivered over a time period of 3 weeks, and is sometimes extended by 1 or 2 weeks (see Table 1 for details).

### Independent variables

Independent variables were 1.) the rate of AGC (OPS 8–550) per hospital and 2.) the rate of TPR per hospital, each calculated as the number of hip fracture patients with an AGC (or a TPR) in each hospital divided by the total number of hip fracture patients in the same hospital. Each variable was categorised into three groups: none (0%), medium (> 0 to ≤ 48.8%) and high (> 48.8%) rates of AGC, and low (< 22.9%), medium (22.9 to 47.1%) and high (> 47.1%) rates of TPR. For AGC the chosen categories followed a dichotomisation (median split: medium and high), and for TPR a tertile split.

The OPS8-550 procedure code and information about transfers to a post-acute rehabilitation clinic were used to characterise the degree to which hospitals provided AGC or TPR for their patients. Whilst, at first sight, it might seem straightforward to base analysis on individual-level data based directly on the presence or absence of OPS8-550 codes or the information about a transfer to a post-acute rehabilitation clinic in individuals’ claims records, this approach is misleading and therefore not appropriate. Reasons are that a) individual allocation on the basis of OPS8-550 coding would introduce immortal time bias (survivorship bias) [20] since to be coded OPS8-550 the patient must receive a minimum of 14 days AGC, and b) that individual allocation on the basis of transfers to a post-acute rehabilitation clinic would introduce a strong selection bias since post-acute rehabilitation is based on functional condition [18]. Identifying the hospitals’ rate of AGC and TPR overcomes these problems. Thus, hospitals’ geriatric health service processes, in general, were used to analyse the association of different types of geriatric treatment with second hip fractures on a systemic level.

### Dependent variables

Outcome was a second hip fracture either after surgery for the index hip fracture (time period: 0 to 180 or 360 days) or after discharge (time period: 6 weeks after surgery for the index hip fracture to 180 or 360 days). For the analysis of the risk of second hip fractures after discharge, 6 weeks as the commencement of follow-up was chosen a) since nearly all patients are discharged after 6 weeks independent of the geriatric treatment system and b) since it guarantees the same observation period for all patients. Second hip fractures were identified using identical criteria and methodology to the index hip fractures in the health claims data.

An additional outcome was new prescriptions of specific anti-osteoporotic drugs (bisphosphonates and denosumab; ATC-codes: M05BA, M05BB, M05BX) prescribed after discharge and dispensed by community pharmacists. Drugs given during hospital stay were not recorded in the dataset and could therefore not be identified for the analyses. For prescriptions of new specific anti-osteoporotic drugs follow-up was restricted to 180 days since prescriptions beyond this time are possibly no longer related to the index hospitalisation. A prescription was defined as ‘new’ if no specific anti-osteoporotic drug was prescribed within 180 days before the index surgery.

We did not analyse Vitamin D prescriptions since Vitamin D needs specific conditions for reimbursement in Germany. Therefore, a large percentage of Vitamin D is dispensed over the counter and not recorded in the health claims data [21].

### Covariates

Age in years at the index fracture and sex were documented in the claims database. An assessment of the degree of care need is a requirement for those identified as „frail care recipients “ under German Social Security Code XI (‘*Sozialgesetzbuch’*). In order to claim for long-term care benefit, people must submit an application and need a daily minimum of 90 min of assistance with basic activities of daily living such as washing, eating, or dressing and of instrumental activities of daily living such as cleaning or shopping. Verification and assessment of care need are performed by the health insurance funds’ medical services. Depending on the extent of care required recipients are categorised into one of three degrees of care need. This classification of care need can be used as a surrogate marker of disability [22]. The number of hip fracture patients per hospital per year in our dataset was used as a surrogate for the size and/or the expertise of the trauma surgery unit. Time to surgery has long been known to be associated with mortality [23]. Therefore, time from hospital admission to surgery was determined and used as a covariate. Because of the link to reimbursement, the coding of comorbidities seemed to be particularly comprehensive in patients with an OPS8-550 procedure. Therefore, common comorbidity scores based on diagnoses might bias the results. Instead, a medication-based co-morbidity score was applied [24]. Prescriptions of medications were coded for the year before fracture and if at least one of the prescribed medications belonged to one of 22 pre-defined disease groups, the co-morbidity counter was increased by one score point.

### Statistical analysis

Baseline characteristics were described by means and standard deviations or absolute numbers and percentages, as appropriate. Cumulative incidences were calculated for study outcomes occurring within 180 and 360 days of initial surgical treatment for a near-hip femur fracture.

The effect of AGC and TPR per hospital on new prescriptions of specific anti-osteoporotic drugs and on second fracture incidence was calculated for each independent variable and mutually adjusted for AGC or TPR.

Hazard ratios (HR) with 95% confidence intervals (CI) were estimated using multivariable Cox proportional hazard models with random effects (clustered on hospitals) to assess the association between AGC or TPR and the incidence of second fractures. Generalised linear models with a Poisson distribution and a log link function were used to estimate incidence rate ratios for new prescriptions of anti-osteoporotic drugs (gComp function in the R package riskCommunicator). The calculation of 95% CI takes into account the clustering of patients within a particular hospital. All models were adjusted for age, sex, need for care on the day before fracture, the number of hip fracture patients per hospital per year, and medication-based comorbidity score. In all models, the continuous variables (age, number of hip fracture patients/hospital/year, medication-based comorbidity score) were standardised to have mean zero and unit variance.

Statistical analyses were performed using R version 4.2.2 (R Foundation for Statistical Computing, Vienna, Austria).

## Results

The analyses included 29,096 patients aged ≥ 80 years from 652 hospitals from 16 federal states which make up the Federal Republic of Germany. The mean age at hospital admission was 87.1 years, and 20.7% were male (Table 2).

**Table 2:** Baseline Characteristics

Hospitals	(*n* = 652)
Rate of acute geriatric care (AGC)^a^	
None; *n* (%)	220 (33.7)
Medium; *n* (%)	216 (33.1)
High; *n* (%)	216 (33.1)
Rate of transfers to post-acute rehabilitation (TPR)†	
Low; *n* (%)	218 (33.4)
Medium; *n* (%)	220 (33.7)
High; *n* (%)	214 (32.8)
**Patients**	(***n*** = 29,096)
Rate of acute geriatric care (AGC)a	
None; *n* (%)	7077 (24.3)
Medium; *n* (%)	12,247 (42.1)
High; *n* (%)	9772 (33.6)
Rate of transfers to post-acute rehabilitation (TPR)†	
Low; *n* (%)	9606 (33.0)
Medium; *n* (%)	10,092 (34.7)
High; *n* (%)	9398 (32.3)
Age (years); Mean (SD)	87.1 (4.6)
Male; *n* (%)	6021 (20.7)
Medication-based comorbidity score (mean (SD))	4.1 (2.0)
Specific anti-osteoporotic drugs 180 days before index surgery	1720 (5.9)
Care need at admission; *n* (%)	19,406 (66.7)
Resident of a nursing home at admission; *n* (%)	7112 (24.4)
Days from hospital admission to index-surgery	
0; *n* (%)	10,533 (36.2)
1; *n* (%)	13,200 (45.4)
2; *n* (%)	3261 (11.2)
≥ 3; *n* (%)	2102 (7.2)
Length of hospital stay (days); Mean (SD)	18.7 (10.8)

^*^Rate of acute geriatric care (OPS 8–550): None: 0%; Medium: > 0 to ≤ 48.8%; High: > 48.8%

^†^Rate of transfer to post-acute rehabilitation: Low: < 22.9%; Medium: 22.9–47.1%; High: > 47.1%

 In 3.6% of patients with hip fracture new prescriptions of specific anti-osteoporotic drugs were dispensed by community pharmacists in the period between discharge and 180 days follow-up (Supplement Table A). No AGC and medium rates of AGC were both associated with lower probabilities of new prescriptions of specific anti-osteoporotic drugs compared to high rates of AGC (Incidence rate ratio (95% CI) 0.60 (0.42–0.89) and 0.49 (0.35–0.67), respectively). Rates of TPR were not associated with the probability of new prescriptions of specific anti-osteoporotic drugs (Table 3).

**Table 3:** Associations of rates per hospital of acute geriatric care (AGC) and transfers to post-acute geriatric rehabilitation (TPR) on new prescription of specific anti-osteoporotic drugs between discharge and 180 days after index surgery in 27,376 patients with hip fracture aged 80 years and older without specific anti-osteoporotic medication in the 180 days before the index surgery

New prescription of specific anti-osteoporotic drugs^&^				
Rate of AGC^*^	***n*** (%)	**IR**	**IRR (95% CI) ‡**	**IRR (95% CI)#**
None	246 (3.7)	9.7	**0.69 (0.49–0.97)**	**0.60 (0.42–0.89)**
Medium	327 (2.8)	7.3	**0.55 (0.39–0.71)**	**0.49 (0.35–0.67)**
High	460 (5.0)	13.1	1.00 (reference)	1.00 (reference)
**Rate of TPR†**				IRR (95% CI)^**^
Low	355 (3.9)	10.3	1.05 (0.75–1.43)	0.73 (0.48–1.03)
Medium	306 (3.2)	8.4	0.81 (0.61–1.09)	0.77 (0.58–1.03)
High	372 (4.2)	10.8	1.00 (reference)	1.00 (reference)

*IR* incidence rate per 100 person-years, *IRR* incidence rate ratio, *CI* confidence interval accounting for clustering of patients within hospital

^*^Rate of acute geriatric care (OPS 8–550): None: 0%; Medium: > 0 to ≤ 48.8%; High: > 48.8%

^†^Rate of transfer to post-acute rehabilitation: Low: < 22.9%; Medium: 22.9–47.1%; High: > 47.1%

^**^Adjusted for age, sex, care need the day before the fracture, number of hip fracture patients/hospital/year, medication-based co-morbidity score, and frequency category of patients per hospital with acute geriatric care

^‡^Adjusted for age, sex, care need the day before the fracture, number of hip fracture patients/hospital/year, and medication-based co-morbidity score

^#^Adjusted for age, sex, care need the day before the fracture, number of hip fracture patients/hospital/year, medication-based co-morbidity score, and frequency category of patients per hospital transferred to post-acute rehabilitation

^&^Bisphosphonates, Denosumab

During follow-up of 180 and 360 days after surgery, 2.9% and 3.9% of the patients with an index hip fracture suffered from a second hip fracture (Supplement Table A).

Treatment in hospitals with AGC was not associated with the risk of a second hip fracture. Treatment in hospitals with low rates of TPR was associated with a 22% and 13% higher risk of second hip fracture within 180 and 360 days compared to treatment in hospitals with high rates of TPR. However, the association did not reach statistical significance (Hazard ratio (95% CI) 1.22 (0.98–1.52) and 1.13 (0.94–1.35), respectively) (Table 4).

**Table 4:** Associations of rates per hospital of acute geriatric care (AGC) and transfers to post-acute geriatric rehabilitation (TPR) on second hip fracture after index surgery (*n* = 29,096) and after discharge (*n* = 25,303) in patients with hip fracture aged 80 years and older

	**Second hip fracture after surgery**					
	**Time period: 0 to 180 days**			**Time period: 0 to 360 days**		
Rate of AGC^*^	***n*** (%)	**HR (95% CI) ‡**	**HR (95% CI)#**	***n*** (%)	**HR (95% CI) ‡**	**HR (95% CI)#**
None	208 (2.9)	1.00 (0.84–1.20)	1.13 (0.91–1.41)	282 (4.0)	1.03 (0.88–1.20)	1.12 (0.93–1.34)
Medium	340 (2.8)	0.94 (0.79–1.11)	1.03 (0.85–1.25)	455 (3.7)	0.95 (0.82–1.09)	1.00 (0.85–1.18)
High	290 (3.0)	1.00 (reference)	1.00 (reference)	387 (4.0)	1.00 (reference)	1.00 (reference)
**Rate of TPR†**			HR (95% CI)^**^			HR (95% CI)^**^
Low	302 (3.1)	1.16 (0.97–1.39)	1.22 (0.98–1.52)	393 (4.1)	1.09 (0.94–1.27)	1.13 (0.94–1.35)
Medium	277 (2.7)	1.01 (0.85–1.20)	1.02 (0.86–1.21)	373 (3.7)	0.99 (0.86–1.13)	0.99 (0.86–1.14)
High	259 (2.8)	1.00 (reference)	1.00 (reference)	358 (3.8)	1.00 (reference)	1.00 (reference)
	**Second hip fracture after discharge**					
	**Time period: 6 weeks to 180 days**			**Time period: 6 weeks to 360 days**		
**Rate of AGCa**	***n*** (%)	**HR (95% CI) ‡**	**HR (95% CI)#**	***n*** (%)	**HR (95% CI) ‡**	**HR (95% CI)#**
None	110 (1.8)	1.13 (0.90–1.43)	**1.35 (1.01–1.80)**	184 (3.0)	1.11 (0.92–1.33)	1.21 (0.97–1.50)
Medium	160 (1.5)	0.95 (0.76–1.18)	1.07 (0.83–1.38)	276 (2.6)	0.94 (0.80–1.11)	1.01 (0.83–1.21)
High	136 (1.6)	1.00 (reference)	1.00 (reference)	236 (2.8)	1.00 (reference)	1.00 (reference)
**Rate of TPR†**			HR (95% CI)^**^			HR (95% CI)^**^
Low	147 (1.8)	1.20 (0.95–1.51)	**1.35 (1.03–1.79)**	241 (2.9)	1.08 (0.90–1.28)	1.15 (0.94–1.41)
Medium	137 (1.6)	1.06 (0.85–1.33)	1.07 (0.86–1.34)	234 (2.7)	1.00 (0.85–1.18)	1.01 (0.85–1.19)
High	122 (1.5)	1.00 (reference)	1.00 (reference)	221 (2.7)	1.00 (reference)	1.00 (reference)

*HR* hazard ratio estimated in a Cox proportional hazards model with random hospital effects (clustered on hospitals), *CI* confidence interval

^*^Rate of acute geriatric care (OPS 8–550): None: 0%; Medium: > 0 to ≤ 48.8%; High: > 48.8%

^†^Rate of transfer to post-acute rehabilitation: Low: < 22.9%; Medium: 22.9–47.1%; High: > 47.1%

^**^Adjusted for age, sex, care need the day before the fracture, number of hip fracture patients/hospital/year, medication-based co-morbidity score, and frequency category of patients per hospital with acute geriatric care

^‡^Adjusted for age, sex, care need the day before the fracture, number of hip fracture patients/hospital/year, and medication-based co-morbidity score

^#^Adjusted for age, sex, care need the day before the fracture, number of hip fracture patients/hospital/year, medication-based co-morbidity score, and frequency category of patients per hospital transferred to post-acute rehabilitation

The effect estimates of the associations between rates of AGC or rates of TPR and second hip fractures increased if only the time after discharge (6 weeks after index-surgery until end of follow-up) was analysed. Treatment in hospitals with no AGC was associated with a higher risk of second hip fractures compared to treatment in hospitals with high rates of AGC (Hazard ratio (95% CI) 1.35 (1.01–1.80)), and treatment in hospitals with low rates of TPR was also associated with a higher risk of second hip fracture compared to treatment in hospitals with high rates of TPR (Hazard ratio (95% CI) 1.35 (1.03–1.79)) if only the time period after discharge (6 weeks after surgery) and 180 days was analysed. The effects attenuated if follow-up was extended to 360 days (Table 4).

## Discussion

In this retrospective cohort study based on nationwide claims data and including over 29,000 patients aged 80 years and over with hip fracture we found hints for an association between low transfer rates to post-acute rehabilitation clinics and an increased risk of second hip fractures. If the time period of analysis was restricted to the first months after discharge, involvement of geriatricians in acute or post-acute care of hip fracture patients was associated with lower risks of second hip fractures. In addition, lower rates of AGC were associated with fewer new prescriptions of specific anti-osteoporotic drugs.

Notably, our results do not describe the risks or benefits for any single individual who receives AGC or is transferred to post-acute rehabilitation. Since both geriatric treatment systems require a pre-defined functional status an approach which analyses the effects of AGC or post-acute geriatric rehabilitation at an individual level would introduce strong selection bias. Instead, we used a systemic approach and analysed the potential benefits of the two different geriatric treatment systems according to their availability in the hospitals in which patients with hip fracture were treated. Therefore, the degree of individual benefit of receiving one or the other geriatric treatment is probably higher than our reported estimates.

There is evidence that organisational structures, like an orthogeriatric co-management or a fracture liaison service, improve post-fracture treatment with anti-osteoporotic drugs [14]. This is in line with our results. There is, however, so far no evidence at all that involvement of acute or post-acute geriatric care is associated with a reduction in second fractures [14]. Our results suggest that acute and post-acute geriatric care may both have a beneficial effect on the incidence of second hip fractures within the first months after hip fracture.

The reasons are speculative. AGC may reduce fall risk by improving physical capacity [25]. In Germany, orthogeriatric care is often delivered in certified Centres for Geriatric Trauma (DGUs) [26] in which standard operating procedures (SOPs) for the management of osteoporosis are mandatory. This may be one explanation for the higher treatment rates with new specific anti-osteoporotic drugs in hospitals with high rates of AGC. However, it is probably not an explanation for the lower fracture risk in the first months after discharge since the observed absolute numbers of new prescriptions were still very low in all analysed treatment groups and since the beneficial effect of anti-osteoporotic drug treatment is expected in the long-term. Post-acute geriatric care, i.e. geriatric rehabilitation, may also reduce fall risk through exercise like strength and balance training and compensating measures like walking aids. Therefore, we recommend that the World Guidelines for Fall Prevention [27] are followed and specifically the following recommendation: “Older adults after sustaining a hip fracture should be offered an individualised and progressive exercise program aimed at improving mobility […] as a fall prevention strategy[…]. Such programs for older adults after a hip fracture are best commenced in hospital […] and continued in the community.”

Strengths of our study are the large number of included hospitals and patients, and the chosen methodical approach to analyse the scientific question on a systemic level instead of an individual level. In our opinion, this approach is robust and conservative and reduces the risk of selection bias considerably. Index fractures and second fractures were identified by a combination of ICD-codes and surgery procedure codes and are therefore thought to be highly reliable even though only based on health claims data.

Our study has some limitations. It is an observational study analysing a heterogeneous situation of geriatric care in Germany. Therefore, any form of bias cannot be completely excluded. Particularly we recognise that there may be biases inherent in routine claims data, for example, services and access to services may not be available uniformly to all older people across Germany, and thus such potential sources of bias must be recalled when interpreting the results. Since we used health claims data we were not able to adjust for potential confounders like cognition or social support. Drugs given during hospitalisation were not recorded in the dataset. Therefore, anti-osteoporotic drugs with low treatment frequencies like zoledronic acid or denusomab are not captured by our analyses if given during hospitalisation. The true number of new anti-osteoporotic drugs is therefore expected to be higher than reported by our study. Furthermore, new treatment with anti-osteoporotic drugs may not have been caused during hospitalisation but initiated by physicians after hospitalisation. It is our experience that physicians usually follow recommendations from hospitals regarding drug treatment after discharge and that initiation of new anti-osteoporotic drugs is rather rare. There is no reason to believe that the degree of underestimation of new anti-osteoporotic drugs differs between the different geriatric treatment systems. Therefore, we expect no systematic bias.

On the one hand, it is the nature of our observational study to analyse associations which may be influenced by the specific structure of the German healthcare system. Naturally, the generalisability of the study results to other countries may therefore be limited. On the other hand, acute geriatric care and post-acute geriatric rehabilitation represent two basic principles of geriatric treatment that are present in many countries of the industrialised world.

## Conclusions

In summary, we analysed the associations of two geriatric treatment systems with second hip fracture in patients aged ≥ 80 years following an index hip fracture. Our study demonstrated that low involvement of geriatricians in the acute or post-acute care of hip fracture patients was associated with an increased risk of second hip fractures in the first months after discharge. Reasons may be the comprehensive treatment and mobilisation by the geriatric team. In addition, new prescriptions with specific anti-osteoporotic drugs were relatively low in all types of hospitals but particularly low in hospitals with low rates of acute geriatric care.

### Supplementary Information

**Supplementary Material 1**

## Data Availability

Data cannot be shared publicly because they originated from the routine data of the health insurance company. For further information, contact Gisela Büchele (gisela.buechele@uni-ulm.de).

## Abbreviations

AGC: Acute geriatric care
TPR: Transfer to post-acute rehabilitation
FRID: Fall-risk-increasing drug
AOK: Allgemeine Ortskrankenkasse
WIdO: Wissenschaftliches Institut der AOK
OPS: Operationen- und Prozedurenschlüssel
ATC code: Anatomical Therapeutic Chemical code
HR: Hazard ratio
CI: Confidence interval
SOP: Standard operating procedure
