# Supplementary material for: Association of two geriatric treatment systems with anti-osteoporotic drug treatment and second hip fracture in patients with an index hip fracture: retrospective cohort study
Source: BMC Geriatr. 2024 May 4;24:395. doi: 10.1186/s12877-024-04989-0 (PMC11069171; doi:10.1186/s12877-024-04989-0)
Supplement: Supplementary file 1 — Supplementary Material 1 [file 12877_2024_4989_MOESM1_ESM.docx]

**Supplement**

Supplement Table A: New prescriptions of specific anti-osteoporotic drugs, second hip fractures and deaths during the different periods of follow-up in patients with hip fracture aged 80 years and older

|  | After index surgery (n=29096) | |  | After discharge (n=25303) | |
| --- | --- | --- | --- | --- | --- |
|  | 1 to 180 days, n (%) | 1 to 360 days, n (%) |  | 6 weeks to 180 days, n (%) | 6 weeks to 360 days, n (%) |
| New prescriptions of AO* drugs | 1033 (3.6) |  |  |  |  |
| Second hip fractures | 838 (2.9) | 1124 (3.9) |  | 406 (1.6) | 696 (2.8) |
| Deaths | 7505 (25.8) | 9451 (32.5) |  | 3712 (14.7) | 5658 (22.4) |

*specific antiosteoporotic drugs (Bisphosphonates, Denosumab)
